# Long-term diagnostic and social outcomes after first psychiatric hospitalization

**DOI:** 10.1192/j.eurpsy.2026.10178

**Published:** 2026-02-27

**Authors:** Julie Nordgaard, Ditte Saebye, Josef Parnas, Peter Handest, Janne Petersen, Sofie Oersted-Hoeyer, Mads Gram Henriksen

**Affiliations:** 1 Psychiatry East, Mid-Western Hospital & Department of Clinical Medicine, University of Copenhagen, Denmark; 2 Center for Clinical Research and Prevention, Bispebjerg and Frederiksberg Hospitals, Denmark; 3 Center for Subjectivity research, University of Copenhagens, Denmark; 4 JALT Psychiatry, Denmark; 5 Center for Clinical Research and Prevention, Bispebjerg and Frederiksberg Hospitals & Section of Biostatistics, Department of Public Health, University of Copenhagen, Denmark; 6 Faculty of Health and Medical Sciences, University of Copenhagen & Mental Health Center Amager, Denmark; 7 Psychiatry East, Mid-Western Hospital & Center for Subjectivity Research, University of Copenhagen: Denmark

**Keywords:** crime, education, mortality, schizophrenia, schizotypy

## Abstract

**Background:**

The early phases of severe mental disorders are often diagnostically challenging, with frequent diagnostic shifts over time. Few studies have combined detailed baseline diagnostic assessment with long-term follow-up to examine both diagnostic and social development.

**Methods:**

We conducted a 20-year register-based follow-up of 150 patients with first-time psychiatric hospitalizations. At baseline, all participants underwent a comprehensive diagnostic assessment. Follow-up data were obtained through linkage to national registers, providing information on psychiatric diagnoses, education, family formation, crime, mortality, and suicide. Cumulative incidence functions accounting for competing risks were calculated stratified on baseline diagnoses.

**Results:**

Only seven participants (4.6%) had no further contact with hospital-based psychiatry during the 20-year follow-up. During the follow-up period, 37.9% received a diagnosis of schizophrenia, 35% schizotypy, 14.4% depression, 24.6% personality disorder, 11% bipolar disorder, and 6.1% substance use disorder. Participants with a baseline diagnosis of schizophrenia, schizotypy, or depression had a significantly higher probability of receiving the same diagnosis during follow-up (schizophrenia 81.6%, schizotypy 69.4%, and depression 53.3%), whereas this was not the case for participants with a baseline diagnosis of personality disorder. Mortality was elevated (5.9%), with suicide accounting for one-third of all deaths, ten times the national average.

**Conclusions:**

A first psychiatric hospitalization in early adulthood marked the beginning of a longer clinical trajectory: 95% of participants re-entered hospital-based care or had prolonged initial hospitalization. The findings emphasize the importance of diagnostic assessment and sustained care to improve prognosis and reduce social impairment and premature death.

## Introduction

The early phases of severe mental disorders are often diagnostically ambiguous, and psychiatric diagnoses frequently change in this period. Longitudinal studies have consistently shown substantial diagnostic shifts, reflecting both the evolving nature of mental disorders and the limitations of current classification systems in capturing prodromal or early-stage presentations [1–3]. Such diagnostic instability has important clinical implications. A diagnosis at first admission may shape treatment decisions, service pathways, and even the patient’s self-understanding, yet its long-term predictive validity is often uncertain. Some diagnostic categories, such as schizophrenia, appear relatively temporally stable [4, 5], while others, including affective or personality disorders, show greater variability [6]. Understanding the long-term diagnostic patterns is essential for improving timely and accurate diagnosis and clinical care but also, ultimately, for refining diagnostic classification systems.

Severe mental disorder is not defined by diagnostic category alone but also by the profound impact on social functioning [7]. Educational disruption, unemployment, difficulties in intimate relationships, and long-term marginalization are common across schizophrenia spectrum disorders and related conditions [7, 8]. As recent work has suggested, such social impairments may not only be secondary consequences of mental disorders but may be structurally embedded in the disorders themselves [7, 8].

To advance our understanding of diagnostic development and long-term prognosis, studies combining in-depth baseline diagnostic assessment with very long follow-up are needed. The present study addresses this need by examining the 20-year diagnostic recurrence/persistence and social outcomes of a cohort of first-admission psychiatric patients, who underwent comprehensive, face-to-face diagnostic evaluations at baseline.

## Aim

The aim of this study was twofold:

1. To examine subsequent diagnoses assigned to a sample of first-admission patients, who underwent a comprehensive diagnostic evaluation at baseline
2. To investigate outcomes of specific social variables in the same group of participants

## Methods

### Sample and baseline assessment

The sample comprised 155 first-admission psychiatric participants, all under the age of 40, consecutively referred to a general psychiatric hospital, serving a catchment area of approximately 130,000 inhabitants in Copenhagen, Denmark, between September 1, 1998, and September 1, 2000.

The purpose of the baseline study was “to explore whether the ‘not-yet-psychotic’ (nondelusional, nonhallucinatory) anomalies of subjective experience aggregate selectively in first-admission patients with schizophrenia spectrum disorder compared to first-admitted patients with diagnoses outside the schizophrenia spectrum” [9] (*p.* 260).

Exclusion criteria in the baseline study were a diagnosis of melancholia, bipolar disorder, or organic brain disorder; primary or clinically dominant substance use; involuntary admission; or forensic status. The reason for excluding melancholia and bipolar disorder was, as stated in the baseline study, that “the incidence of these conditions was a priori expected to be too low for arriving at a statistically informative sample size within the study’s time limit” [9] (*p.* 260). Patient with organic disorders (coarse brain disorder and clinically dominant substance use disorders) were excluded as the psychopathology was most likely caused by the organic disorder or substance use and thus not relevant to the baseline study. Finally, patients with forensic status were not included due to ethical concerns (it can be discussed if you feel completely free to reject participation in a research study if you are admitted to the hospital against your will).

Primary or clinically dominant substance use disorder, defined as cases in which substance use or a substance-induced mental disorder fully accounted for the presenting condition, was an exclusion criterion at baseline. Comorbid substance use disorder was permitted when an independent psychiatric disorder was clinically established.

Severely psychotic or aggressive individuals were interviewed only after clinical stabilization. All participants provided written informed consent.

Four individuals were subsequently excluded due to a verified organic psychiatric disorder, resulting in a final sample of 151 participants (for details, see [10]).

At baseline, all participants underwent a comprehensive, face-to-face, semi-structured, clinical interview conducted by an experienced consultant psychiatrist. The interview lasted 3–5 hours and included a detailed assessment of family history (with input from secondary informants when available), psychosocial history, and a diagnostic evaluation using the Operational Criteria Checklist for Psychotic Illness (OPCRIT) [11], expanded with selected items from the Copenhagen High Risk Study [12]. A phenomenological exploration of subjective anomalies was assessed using the Danish version of the Bonn Scale for the Assessment of Basic Symptoms (BSABS) [13], supplemented with items targeting self-disorders [14]. Additional instruments included the Positive and Negative Syndrome Scale (PANSS) [15], and the Premorbid Adjustment Scale (PAS) [1].

All ICD-10 [16] research diagnoses were established based on the full baseline interview material and available collateral information. Diagnostic consensus was reached between the interviewer (author PH, who at the time of inclusion was an experienced psychiatrist and researcher) and a senior psychiatrist (author JP, who at the time of inclusion was a clinical professor of psychiatry with extensive clinical experience). In cases of diagnostic disagreement, the clinically treating psychiatrist was consulted and a consensus diagnosis was reached. Inter-rater diagnostic reliability between PH and JP was high, with a Cohen’s kappa of 0.80 [17, 18].

### Register-based follow-up

A 20-year follow-up was conducted using data from national Danish registers at Statistics Denmark. Each patient was linked via their unique personal identification number to the following Danish registries: The National Patient Register [19] to identify psychiatric diagnoses recorded in inpatient or outpatient settings; The Cause of Death Register [20] to obtain information on vital status and cause of death; National registries to obtain information on the highest level of attained education, crime, and socio-demographic data [21].

The follow-up period was set to 3 months after the baseline assessment, and it was extended to 20 years post-baseline. One of the 151 patients could not be included in the follow-up study because a personal identification number was not available, which precluded linkage to the registers. Thus, 150 participants were included in the 20-year follow-up, retrospective cohort study. Diagnoses from the National Patient Register were categorized into ICD-10 diagnostic groups. Social variables included parenthood, criminal convictions, and education. Mortality data were available for a longer follow-up period (21.2 years) than the other outcome measures.

Baseline diagnoses were divided into the following six categories: Schizophrenia (ICD-10: F20.0-F20.9); Schizotypy (ICD-10: F21); Depression (ICD-10: F32.0-F33.9); Personality Disorder (ICD-10: F60.0-F69.9); Substance Use Disorder (ICD-10: F10.0–19.9); and Other Mental Illnesses (OMI), which included all diagnoses not fitting the other diagnostic categories, including delusional disorder and brief psychotic disorder.

The same diagnostic categories and, in addition, bipolar disorder (ICD-10: F31.0–31.9), which was an exclusion criterion for the initial study, were applied for the follow-up diagnoses. Due to Danish legislation, it is not possible to report information for diagnostic categories or any other information with fewer than four individuals.

### Statistical analyses

To estimate the 20-year cumulative incidence of the outcome in the presence of competing risks (e.g., death), we used the PROC LIFETEST procedure in SAS Enterprise Guide. The presence or absence of each of the diagnoses of interest at baseline stratified the analyses. Survival time was defined as from three months after baseline to the occurrence of the event or censoring. Individuals were censored at the end of follow-up, on 31.12.2020, due to migration, or death from causes other than the event of interest. To formally compare the cumulative incidence between groups (diagnosis present versus absent), Gray’s test was performed. This test evaluates whether the cumulative incidence functions differ significantly between strata in the presence of competing risks.

## Results

### Sample characteristics at baseline

One hundred fifty first-admission patients were included. Baseline characteristics for both the whole sample and stratified into diagnostic groups are presented in Table 1.

**Table 1.** Baseline descriptives

Baseline diagnosis→Baseline variables ↓	Schizophrenia	Schizotypy	Depression	Personality disorder	Substance use disorder	OMI^a^	All	*p*-value^b^
*N* (%) in diagnosis group	41 (27.3%)	50 (33.3%)	15 (10%)	30 (20%)	6 (4%)	8 (5.3%)	150	
*N* (%)								
Males	23 (56.1%)	14 (28.0%)	6 (40.0%)	8 (26.7%)	Not reported	Not reported	57	
Females	18 (43.9%)	36 (72.0%)	9 (60.0%)	22 (73.3%)	Not reported	Not reported	93	0.0320
Married/living with a partner for 6 months or more	14 (34.1%)	24 (48.0%)	6 (40%)	21 (70.0%)	Not reported	Not reported	N diff too small^c^	0.0158
Unmarried/living without a partner/unknown	27 (65.9%)	26 (52.0%)	9 (60.0%)	9 (30.0%)	Not reported	Not reported	N diff too small^c^	
Working	Not reported	Not reported	Not reported	Not reported	Not reported	Not reported	140	0.1479
Not working	Not reported	Not reported	Not reported	Not reported	Not reported	Not reported	10	
Not social isolated	14 (34.1%)	21 (42.0%)	Not reported	23 (76.7%)	Not reported	Not reported	*N* diff too small^3^	<0.0001
Social isolated	27 (65.9%)	29 (58.0%)	Not reported	7 (23.3%)	Not reported	Not reported	*N* diff too small^c^	
No alcohol- or substance- use disorder	31 (75.6%)	37 (74.0%)	Not reported	20 (66.7%)	Not reported	Not reported	111	0.3293
Alcohol- or substance- use disorder	10 (24.4%)	13 (26.0%)	Not reported	10 (33.3%)	Not reported	Not reported	39	
Mean (standard deviation)								*p*-value^d^
Age at interview	25.7 (4.5)	25.1 (4.4)	25.2 (3.9)	27.1 (5.0)	27.3 (7.5)	27.1 (5.4)	25.9 (4.7)	0.459
Perceptual disorder at baseline^e^	2.0 (1.9)	1.6 (1.7)	0.7 (1.1)	0.7 (0.9)	1.5 (1.6)	0.8 (0.7)	1.4 (1.6)	0.005
Self-disorders at baseline	6.5 (3.9)	6.0 (2.9)	3.1 (3.4)	2.1 (2.9)	3.7 (5.8)	2.4 (2.4)	4.8 (3.8)	0.000
PANSS positive symptoms at baseline	19.9 (5.2)	11.9 (3.1)	8.5 (1.3)	9.4 (2.6)	13.5 (7.1)	13.5 (7.2)	13.4 (5.8)	0.000
PANSS negative symptoms at baseline	18.5 (5.6)	13.2 (4.0)	11.7 (4.5)	9.1 (2.2)	9.0 (2.9)	10.3 (2.9)	13.4 (5.5)	0.000

a Other mental illness (OMI) includes all other mental disorders (defined as diagnoses from chapter F in the ICD-10, except the exclusion diagnoses, which were organic brain disorder, bipolar disorder and depression with melancholia). No patients were diagnosed with autism (F90.0–90.9) or hyperkinetic disorder (F84.1 + F84.5 + F84.7) at baseline.

b *p*-value from Fishers Exact test for independence between categories baseline variable and baseline diagnosis group.

c Numbers below four cannot be reported, due to protection of person-sensitive information. This also means, that it should not be possible to calculate if a number in a cell is below three. In some situations, this makes it necessary to delete the total *N* for a row.

d *p*-value from non-parametric Kruskal–Wallis Equality Test of the six diagnosis groups.

e Selected BSABS items.

Looking at educational level at baseline, 54 (36.5%) patients had 10 or less years of education, 74 (50%) had between 10 and 13 years, 14 (9.5%) had between 13 and 16 years whereas 6 (4.1%) had more than 16 years of education. There was no information on the years of education for two patients.

### Diagnostic development

Seven individuals did not come into contact with psychiatry and did not receive a subsequent psychiatric diagnosis during the 20-year follow-up period. Thus, 143 participants either had at least one additional contact with hospital-based psychiatry and received a subsequent psychiatric diagnosis or had a first-admission that extended beyond 3 months. Several participants received more than one subsequent diagnosis of a mental disorder.

Overall, the probability that participants received a diagnosis of schizophrenia during follow-up was 37.9%, 35% for a diagnosis of schizotypy, 11% for bipolar disorder, 14.4% for depression, 24.6% for personality disorder, and 6.1% for substance use disorder.

Table 2 presents the cumulative incidence of receiving selected diagnoses at least once during the 20-year follow-up period, stratified by baseline diagnostic group.

**Table 2.** Probability (cumulative incidence) for 1. event register diagnosis, 3 months to 20 years after baseline, divided into whether the patient at baseline had the diagnosis in the first column or not

1. Event of diagnosis after 3 months →Baseline diagnosis ↓	Cumulative incidence CIF (95%-Confidence Interval) in %								
	N (%)	Schizophrenia	Schizotypy	Bipolar	Depression	Personality disorder	Substance use dis.	OMI^&^
All patients included at baseline	150 (100.0)	37.9 (30.1;45.7)	35.0 (27.4;42.7)	11.0 (6.6;16.7)	14.4 (9.3;20.6)	24.6 (17.9;31.8)	6.1 (3.0;10.8)	44.5 (36.3;52.4)
Not Schizophrenia	109 (72.7)	**21.6 (14.3;29.8)**	**44.5 (34.9;53.6)**	12.3 (6.9;19.4)	16.1 (9.8;23.8)	29.2 (20.8;38.0)	5.7 (2.3;11.2)	**50.1 (40.1;59.2)**
Schizophrenia	41 (27.3)	**81.6 (64.7;91.0) *p* = 0.000**	**9.8 (3.1;21.2) *p* = 0.000**	*n* ≤ 4, *p* = 0.384	9.9 (3.1;21.4) *p* = 0.571	12.4 (4.5;24.6) *p* = 0.067	*n* ≤ 4, *p* = 0.701	**29.9 (16.5;44.4) *p* = 0.022**
Not Schizotypy	100 (66.7)	41.3 (31.5;50.8)	**18.0 (11.2;26.2)**	12.4 (6.8;19.8)	13.3 (7.4;20.8)	23.3 (15.5;32.1)	8.1 (3.8;14.6)	39.9 (30.1;49.5)
Schizotypy	50 (33.3)	31.0 (18.5;44.3) *p* = 0.137	**69.4 (54.1;80.5) *p* = 0.000**	8.3 (2.6;18.2) *p* = 0.490	16.6 (7.7;28.5) *p* = 0.795	27.1 (15.4;40.2) *p* = 0.863	*n* ≤ 4, *p* = 0.141	54.0 (38.7;67.0) *p* = 0.140
Not personality dis.	120 (80.0)	45.7 (36.5;54.4)	35.4 (26.8;44.0)	11.1 (6.2;17.6)	17.9 (11.5;25.3)	22.1 (15.1;30.0)	5.9 (2.6;11.2)	45.1 (35.9;53.9)
personality dis.	30 (20.0)	*n* ≤ 4, *p* = 0.000	33.3 (17.2;50.3) *p* = 0.963	*n* ≤ 4, *p* = 0.963	*n* ≤ 4, *p* = 0.013	34.4 (17.7;51.8) *p* = 0.157	*n* ≤ 4, *p* = 0.828	41.0 (22.9;58.3) *p* = 0.982
Not depression	135 (90.0)	40.7 (32.3;49.0)	35.9 (27.8;44.0)	**8.4 (4.4;13.9)**	**10.0 (5.6;15.9)**	22.8 (16.0;30.3)	5.3 (2.3;10.1)	45.0 (36.2;53.3)
depression	15 (10.0)	*n* ≤ 4, *p* = 0.049	26.7 (7.7;50.5) *p* = 0.412	**33.3 (11.5;57.3) *p* = 0.005**	**53.3 (24.9;75.3) *p* = 0.000**	40.0 (15.6;63.6) *p* = 0.128	*n* ≤ 4, *p* = 0.234	40.0 (15.6;63.6) P = 0.474

*Note*: All the *p*-values stem from Gray’s Test for Equality of Cumulative Incidence Functions (CIF) in the two groups Not diagnosis vs diagnosis at baseline, where diagnosis is schizophrenia, schizotypy, personality disorder or depression. CIF, 95%-CI and *p*-values in **bold** are the **significant** CIF with not to small cell counts.

OMI (other mental illness)-category can contain ICD10-diagnoses codes that are not F20.0-F20.9, F21.0-F21.9, F31.0-F33.9, F60.0-F69.99, F84.1 + F84.5, F90.0-F90.9, F10.0-F19.9.

#### Schizophrenia

Among participants diagnosed with schizophrenia at baseline, the probability of receiving a diagnosis of schizophrenia again during the follow-up period was 81.6%. By contrast, the probability of receiving a schizophrenia diagnosis was only 21.6% for those not diagnosed with schizophrenia at baseline (*p* < 0.001).

A baseline diagnosis of schizophrenia was associated with a significantly lower likelihood of receiving a later diagnosis of schizotypy (9.8% vs. 44.5%, *p* < 0.001) compared with those not diagnosed with schizophrenia at baseline. A baseline diagnosis of schizophrenia was also associated with a lower probability of receiving a subsequent diagnosis within the broad category of “Other Mental Disorders” (OMI) (29.9% vs 50.1%, *p* = 0.022). By contrast, a baseline diagnosis of schizophrenia did not significantly affect the likelihood of later receiving a diagnosis of bipolar affective disorder, depression, personality disorder, or substance use disorder.

#### Schizotypy

Participants diagnosed with schizotypy at baseline had a significantly higher probability of receiving the same diagnosis again during follow-up than participants not diagnosed with schizotypy at baseline (69.4% vs. 18.0%, *p* = 0.000). No significant associations were observed between baseline schizotypy and any other diagnostic category.

#### Depression

Participants diagnosed with depression at baseline had a significantly higher likelihood of later being diagnosed with depression (53.3% vs 10.0%, *p* = 0.000) or bipolar disorder (33.3% vs 8.4%, *p* = 0.005) compared to those not diagnosed with depression at baseline.

#### Personality disorder

A baseline diagnosis of personality disorder was not significantly related to a later diagnosis of personality disorder, bipolar disorder, or schizotypy, but it seemed to be associated with a lower likelihood of a later diagnosis of depression or schizophrenia. Yet, the numbers were very low in these categories.

**Figure 1.**
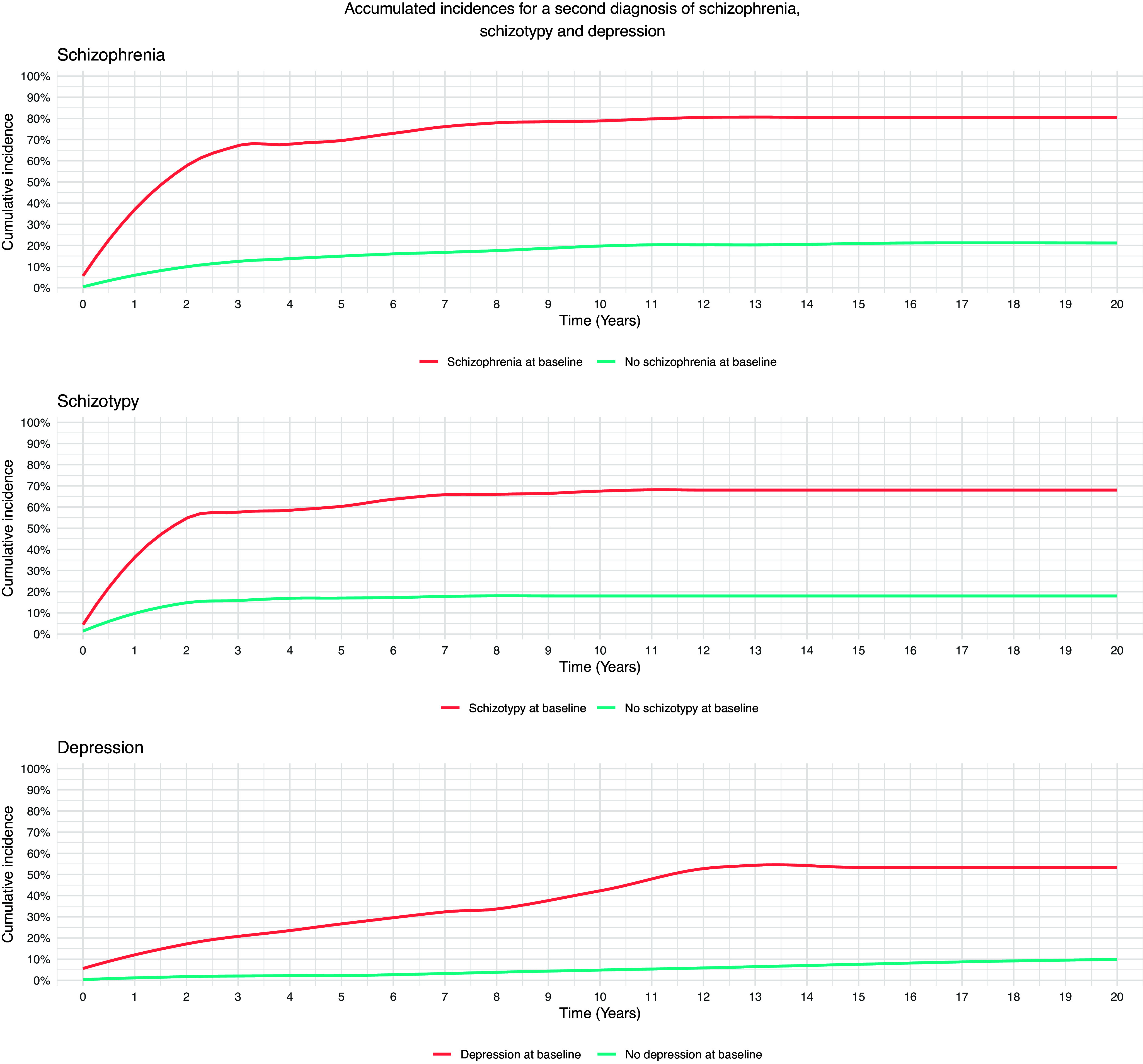
Accumulated incidences for a second diagnosis of schizophrenia, schizotypy and depression.

As being diagnosed with either schizophrenia, schizotypy, or depression significantly increased the likelihood of receiving the same diagnosis again, we display the accumulated incidence curves in Figure 1. Most second schizophrenia- or schizotypy-diagnoses were allocated within the first few years, illustrated with steep slopes in the first 2–3 years. For depression, the slope is less steep in the first 11 years, indicating a more even distribution of the time between the first and second diagnosis of depression.

### Social outcomes

Baseline educational levels were not stratified by diagnostic group because the number of patients in several groups was too small. For the whole sample, many participants pursued further education over the 20-year follow-up time, resulting in the following distribution at follow-up: ≤10 years (lower secondary), 32 participants (21.6%); 10–13 years (upper secondary), 56 (37.8%); 13–16 years (short-cycle/Bachelor), 34 (23.0%); and > 16 years (master), 26 (17.6%). Compared with baseline, 22 of the 54 participants (40.7%) with ≤10 years of education advanced to a higher level, 31 of 74 (41.9%) with 10–13 years advanced beyond upper secondary, and 5 of 14 (35.7%) with 13–16 years attained >16 years of education.

Table 3 shows that the probability of 43% for having children. The probability of being convicted for a crime was 3.4%. 21.2 years after baseline, 5.9% had died, 2.4% by suicide.

**Table 3.** Probability (cumulative incidence) for social variables

Variable	Baseline*N* (%)	Probability of first event in 20 years after baseline***CIF (95%-confidence interval) in %
Part of population	Total *N* = 150	
Children	12 (8%)	Probability of getting a child in 20 years among those without children at baseline: 43% (35;51)
Crime*	≤4^a^	Incidence of criminality in 20 years after baseline among all the 150 probands independently of former crime status: 3.4% (1.3;7.3)
Death^b^	Not relevant	Dies during 20 years after baseline: 4.8% (2.1;9.2) Dies during 21.2 years after baseline: 5.9% (2.7;10.9)
Suicide**	Not relevant	Suicide during 21.2 years after baseline: 2.4% (0.6;6.5)

* Criminal offenses committed within the 5 years preceding baseline.

** We only have information about cause of death up until 31.12.2019.

*** Death is a competing event; censoring occurs at migration or on 31.12.2020.

a Number too small to be given exact.

b Deaths post-emigration are not classified as death events in this table.

## Discussion

This 20-year follow-up study of first-admission psychiatric patients has three main findings. First, in most cases a psychiatric hospitalization in early adulthood signaled the beginning of a long-lasting clinical trajectory: fewer than 5% of our cohort had no further contact with hospital-based psychiatry during the subsequent two decades. Second, while psychiatric diagnoses often change over time, certain diagnostic categories, most notably schizophrenia, had a high probability of being diagnosed again during the follow-up period. Third, enduring social impairments characterize the life course of individuals diagnosed with a mental disorder in early adulthood, and excess mortality, particularly by suicide, underscores the need for continued monitoring and care for this group.

### Diagnostic development

Our findings highlight both the possibilities and limits of early psychiatric diagnosis. Four out of five patients diagnosed with schizophrenia at baseline were rediagnosed with schizophrenia during the 20-year follow-up period, demonstrating the importance of a comprehensive diagnostic assessment at baseline. Participants with a baseline diagnosis of schizotypy or depression also had a significantly higher probability of being rediagnosed with those disorders during follow-up. By contrast, a baseline diagnosis of personality disorder did not have a higher probability of being rediagnosed during follow-up, suggesting either genuine clinical change in this group or more diagnostic variability in routine practice for this group.

These results seem consistent with long-term studies, showing that schizophrenia and bipolar disorder tend to exhibit the greatest stability, while other psychotic disorders are more diagnostically fluid [22–24]. The finding that just above 20% of participants not initially diagnosed with schizophrenia were later given this diagnosis illustrates the enduring challenge of recognizing prodromal or early-stage cases, even after detailed clinical interviews.

The diagnostic stability of schizotypy is a particular strength of the study. Given that schizotypy is often diagnostically unstable in routine clinical practice, the observed persistence over two decades suggests that it represents a meaningful and enduring schizophrenia spectrum condition when carefully assessed at baseline.

Our finding that participants with a baseline diagnosis of personality disorder did not exhibit a significantly higher probability of being rediagnosed with a personality disorder during follow-up is somewhat surprising, given that personality disorders in ICD-10 are defined on the basis of “enduring” and “pervasive” abnormal behavior patterns. Yet, this finding could be mirroring, to some extent, findings from the McLean Study of Adult Development, where a longitudinal follow-up study revealed considerable remission and diagnostic fluctuation in borderline personality disorder [25]. Another study found that only 24% of adolescents with borderline personality disorder met criteria for this diagnosis at 5-year follow-up [26]. Finally, a meta-analysis [6], exploring the diagnostic stability of personality disorder and borderline personality disorder specifically, found that approximately half of the participants were rediagnosed with any personality disorder (56.7%) or borderline personality (45.2%), respectively. This suggests that some diagnostic categories, like personality disorders, may be more sensitive to change over time or variability in clinical practice.

The high probability of diagnostic recurrence/persistence for certain baseline diagnoses, especially schizophrenia, could, in part, reflect diagnostic anchoring, i.e., a cognitive bias whereby clinicians adhere to initial diagnostic impressions, even in the face of evolving clinical evidence [27]. This bias, closely related to what has been termed clinical conservatism, may contribute to apparent diagnostic stability over time [28]. Yet, the absence of a significantly higher probability of being rediagnosed with other mental disorders, particularly personality disorders, makes a purely anchoring-based explanation of our findings untenable.

An important consideration is that the baseline assessments were conducted in 1998–2000. However, ICD-10 has been consistently used in Danish psychiatry since 1994, and throughout the follow-up period, which limits the impact of diagnostic system changes on the observed long-term diagnostic recurrence/persistence. Yet, even within a stable diagnostic system, some changes in clinical awareness, diagnostic practice, or service organization are likely to have occurred.

Importantly, changes in diagnosis over time should not necessarily be interpreted as diagnostic inconsistency or error. Psychiatric diagnoses represent the best clinical formulation based on the information available at a given time, and diagnostic revisions may reflect the emergence of new symptoms, longitudinal clarification of psychopathology, or changing clinical priorities rather than incorrect initial assessment. In some cases, diagnostic change may also reflect the recognition of co-occurring conditions rather than the replacement of one diagnosis by another. However, the present findings suggest that diagnostic stability is not merely a function of administrative persistence or co-occurrence but is closely related to the quality and depth of the initial clinical evaluation. A careful, comprehensive baseline assessment may enable earlier identification of clinically relevant psychopathological features, thereby increasing diagnostic accuracy from the outset and reducing the need for subsequent diagnostic revision.

### Social outcomes

From a social functioning perspective, many participants achieved new social “milestones”: after 20 years, 43% had children. However, approximately 80% of the Danish population has children by the age of 50 [29], pointing to the figures falling well below the population average. In Denmark, approximately 49% of the population had 16 or more years of education in 2020 [30] the proportion was substantially lower in our patient sample, further underscoring persistent educational and social disadvantages. By contrast, the most basic educational level appears to be similar to that of the general population; in 2021, 20% of the Danish population aged 45 or older had 10 years or less of education [31]. Collectively, these patterns of social development seem to reflect enduring functional impairment and sustained socioeconomic disadvantage for a substantial portion of the cohort. While some individuals achieved key social milestones, the overall picture suggests limited social recovery at the population level.

### Mortality and suicide

The mortality rate in this cohort, particularly suicide deaths at 10 times the population average, is consistent with previous evidence of excess mortality in psychiatric populations (Harris & Barraclough 1998; Nordentoft et al. 2011; 2013). These findings highlight the critical importance of sustained monitoring, treatment, and support, especially during long courses of illness.

### Strengths and limitations

A major strength of this study is the combination of rigorous and detailed baseline diagnostic assessments, conducted by experienced psychiatrists, and a nearly complete 20-year follow-up via several national registers. Limitations include a limited sample size and the absence of clinical reassessments during follow-up, constraining insights into symptom trajectories, treatment effects, and reasons for lack of long-term diagnostic recurrence/persistence. Register diagnoses may also reflect administrative practices as much as clinical reality. Furthermore, the baseline exclusion of patients with primary substance use, bipolar disorder, or melancholia at baseline limits the generalizability of the findings.

Although the cohort was recruited consecutively from a general psychiatric inpatient setting with the intention of including a broad range of first-admission patients, the resulting diagnostic distribution reflects both the inclusion and exclusion criteria and the clinical characteristics of patients admitted to inpatient psychiatry at the time. Consequently, schizophrenia spectrum disorders were relatively frequent in the sample. This enrichment was not intentional, but should be considered when interpreting diagnostic stability and long-term diagnostic recurrence/persistence.

Patients with forensic status were excluded for ethical reasons, which may also have influenced the diagnostic composition of the cohort. Under Danish legislation, involuntary admission is typically contingent on the presence of a psychotic state; inclusion of forensic patients would therefore likely have increased the proportion of patients with psychotic disorders at baseline.

Social outcomes were assessed using register-based indicators such as educational attainment and becoming a parent. While these measures are robust and relevant, they represent limited proxies for social functioning and do not capture important dimensions such as quality of relationships, occupational functioning, or everyday social functioning. This is an inherent limitation of register-based studies. In the Danish context, cohabitation without marriage is common, but not registered thus, we did not include marital status in the register follow-up.

## Conclusion

Taken together, our findings show that a first psychiatric admission in early adulthood often marks a lifelong turning point, with consequences for diagnosis, social functioning, and mortality. While a considerable proportion of participants not diagnosed with schizophrenia at baseline later received this diagnosis, baseline diagnoses of schizophrenia and schizotypy had a higher probability of being rediagnosed during follow-up than other diagnostic categories. In parallel, participants exhibited poorer social functioning and higher mortality rates compared to the general population. Notably, 95% of the cohort either had prolonged initial admissions (>3 months) or re-entered hospital-based psychiatric care during follow-up. These findings underscore the importance of early psychiatric admissions as a critical window of opportunity, not only for diagnostic clarification but also for sustained engagement to prevent social exclusion and premature death.

## Data Availability

The data used in this study are not publicly available due to restrictions related to data protection and privacy regulations.
